# Long‐Term Efficacy of Immunotherapy in Autoimmune Autonomic Ganglionopathy—A 10‐Year Follow Up Study

**DOI:** 10.1002/acn3.70421

**Published:** 2026-05-15

**Authors:** Giacomo Chiaro, Shiwen Koay, Gordon T. Ingle, Patricia McNamara, Laura Watson, Fion Bremner, Valeria Iodice

**Affiliations:** ^1^ Autonomic Unit National Hospital Neurology and Neurosurgery London UK; ^2^ Queen Square Centre for Neuromuscular Diseases UCL Queen Square Institute of Neurology London UK; ^3^ Department of Neuro‐Ophthalmology National Hospital for Neurology and Neurosurgery London UK; ^4^ Department of Translational Neuroscience and Stroke UCL Queen Square Institute of Neurology London UK; ^5^ NIHR University College London Hospitals Biomedical Research Centre London UK

**Keywords:** autoimmune autonomic ganglionopathy, immunotherapy, treatment outcomes

## Abstract

**Objective:**

Autoimmune autonomic ganglionopathy (AAG) is a rare but potentially treatable cause of severe autonomic failure. Evidence guiding long‐term immunotherapy, treatment sequencing, and residual autonomic impairment is limited. We evaluated long‐term treatment response, residual autonomic dysfunction, and relapse patterns in patients with seropositive AAG.

**Methods:**

Patients with seropositive AAG undergoing longitudinal follow‐up with repeated quantitative autonomic assessments before and after immunotherapy were included. Residual autonomic impairment was assessed using cardiovascular, pupillary, and urinary autonomic biomarkers, need for anti‐hypotensive medications or catheterisation, and patient‐reported symptoms measured by the COMPASS‐31 questionnaire.

**Results:**

Of 18 patients with seropositive AAG, 16 had longitudinal quantitative autonomic testing, antibody titres, COMPASS‐31 scores, and documented medication and catheter use before and after immunotherapy. At baseline, all 16 had widespread autonomic failure. All received plasma exchange, 10 received intravenous immunoglobulins, and 11 were treated with oral prednisolone followed by steroid‐sparing agents. Treatment was associated with significant improvements in cardiovascular autonomic markers, antibody titres, and pupillary light responses, with non‐significant improvements in COMPASS‐31 scores. Four patients discontinued catheterisation. Two patients were refractory to plasma exchange and intravenous immunoglobulins but responded to prednisolone. Two patients relapsed while receiving steroid‐sparing immunotherapy. High antibody titres correlated with the severity of cardiovascular and urinary dysfunction at disease onset, but not during follow‐up.

**Interpretation:**

Prolonged stepwise immunosuppression in seropositive AAG is associated with sustained objective autonomic improvement, although residual impairment and relapse remain common. These findings support individualised, biomarker‐guided immunotherapy and highlight the need for ongoing supportive management of autonomic complications.

## 1 Introduction

Since its first descriptions, autoimmune autonomic ganglionopathy (AAG) has become an increasingly recognised but treatable disease presenting with autonomic failure [1, 2]. It is now established that ganglionic acetylcholine receptor auto‐antibodies (gAChR‐Ab) are pathogenic and detected in about 50% of patients with AAG [3, 4].

The original description of AAG typically included previously healthy patients, with a slight female preponderance, presenting with an acute or subacute onset of widespread autonomic failure characterised by neurogenic orthostatic hypotension and equally prominent cholinergic involvement with gastrointestinal dysmotility, sicca syndrome, anhidrosis, pupillary fatigue [5], urinary retention, and erectile dysfunction in males, whose severity at onset correlated with antibody levels [6, 7, 8, 9]. Seronegative cases were described later, suggesting that other unidentified immunologic mechanisms may be involved [3, 10, 11, 12, 13]. The gAChR‐Ab can be detected together with other antibodies in paraneoplastic autonomic neuropathies, mimicking AAG [12, 14].

Patients with AAG can present with either a monophasic, relapsing–remitting, or a slowly progressive course [15]. Sometimes it can appear with a more insidious onset, making it virtually indistinguishable from the alpha‐synucleinopathy pure autonomic failure [16].

Treatment response in AAG is variable, likely reflecting delayed diagnosis, heterogeneity in autonomic involvement, different treatment protocols, and patient comorbidities. Case reports and series show that both seropositive and seronegative patients can experience temporary improvements with plasma exchange (PLEX) or intravenous immunoglobulins (IVIg), though most require longer‐term immunosuppression for sustained disease control [17, 18, 19, 20, 21, 22, 23, 24]. Symptomatic treatment of autonomic dysfunction, including vasopressors for orthostatic hypotension, prokinetics for gastrointestinal dysmotility, catheterisation, and interventions for sexual dysfunction are often needed chronically [25].

While a recent overview highlighted current challenges in the management of AAG and offered practical recommendations to guide treatment decisions [26], the optimal therapeutic regimen is so far unknown, and treatment consensus is lacking. Long‐term outcomes in AAG remain unclear, with no large longitudinal data on symptom burden or gAChR‐Ab titre evolution after treatment. Using a multimodal quantitative autonomic assessment, we have shown that meaningful clinical, functional, and morphological improvement is achievable even in longstanding disease with intensive immunotherapy [25].

In this work, we characterise autonomic dysfunction and long‐term immunotherapy outcomes in a cohort of seropositive AAG patients at our National Autonomic Centre, quantify residual impairment using multimodal autonomic biomarkers, outline a monitoring‐based treatment protocol, and update our previously reported cohort.

## 2 Materials and Methods

### 2.1 Patients

We identified patients with a diagnosis of seropositive AAG referred to the national autonomic tertiary referral centre at the National Hospital for Neurology and Neurosurgery, London, UK, from 2005 to 2024. Consent was obtained according to the Declaration of Helsinki. The study was approved by the local Research Ethics Committee and Health Research Authority.

### 2.2 Ganglionic AChR Antibody Testing

Ganglionic AChR‐Ab levels were measured by a radioimmunoprecipitation assay (RIA) using solubilised antigen from a human neuroblastoma (IMR‐32) cell line bound to ^125^I‐epibatidine as previously described [4], and performed by the University of Oxford Neuroimmunology laboratory since 2005 (reference range: > 100 pmol/L positive titre). Since June 2023, samples are processed with a new, similar commercial RIA kit by RiaRSR gAChR‐Ab, Cardiff, UK (new reference ranges: < 10 pmol/L negative, 10–14 pmol/L equivocal, > 15 pmol/L positive titre).

### 2.3 Quantitative Multimodal Autonomic Function Testing

Beat‐to‐beat measurements of blood pressure and heart rate were recorded and analysed with Labchart 8 Pro software (AD Instruments), as previously described [27]. Neurogenic orthostatic hypotension (nOH) was defined by a drop of ≥ 20 mmHg in systolic blood pressure (SBP) or of ≥ 10 mmHg in diastolic blood pressure within 3 min of active standing test (AST) or head‐up tilt (HUT), absence of the phase‐IV blood‐pressure overshoot after the release of the Valsalva manoeuvre (VM) strain and post‐VM pressure recovery time > 4 s [28]. Abnormal values were defined according to age ranges as previously described [27]. The orthostatic intolerance ratio (OIR) [25] was calculated by dividing the change in systolic blood pressure in mmHg by the maximum time tolerated on tilt in minutes. If a patient tolerated a full 10 min of tilt, 10 was used as the denominator for the calculation.

Baseline pupillary dark diameters and responses to stimulation with white light and topical pharmacological agents were recorded with a custom built infrared pupillometer, as previously described [5, 29, 30].

Urinary flow when voiding with the sensation of a full bladder was assessed by uroflowmetry (Albany Medical SmartFlow) and post‐void residual (PVR) volume measured using a bladder ultrasound scanner (Bardscan Realtime).

### 2.4 Questionnaires

The Composite Autonomic Symptom Score (COMPASS)‐31 was used to assess orthostatic intolerance, vasomotor, secretomotor, gastrointestinal, urinary, and pupillomotor symptoms [31]. This instrument has 6 subscale‐weighted scores in the following domains: orthostatic (4 items; range, 0–40), vasomotor (3 items; range, 0–5), secretomotor (4 items; range, 0–15), gastrointestinal (12 items; range, 0–25), bladder (3 items; range, 0–10), and pupillomotor (5 items; range, 0–5). Total pre‐immunotherapy and post‐immunotherapy scores were calculated by summation of the individual item scores, with a possible maximum score of 100.

### 2.5 Diagnosis, Treatment, and Surveillance Protocol

Since 2016, we have developed a protocol for the diagnosis, treatment, and monitoring of patients with AAG. In 2020, the NHS England IVIg commissioning guidelines were updated to include AAG within the diseases for which IVIg is routinely commissioned.

**FIGURE 1:**
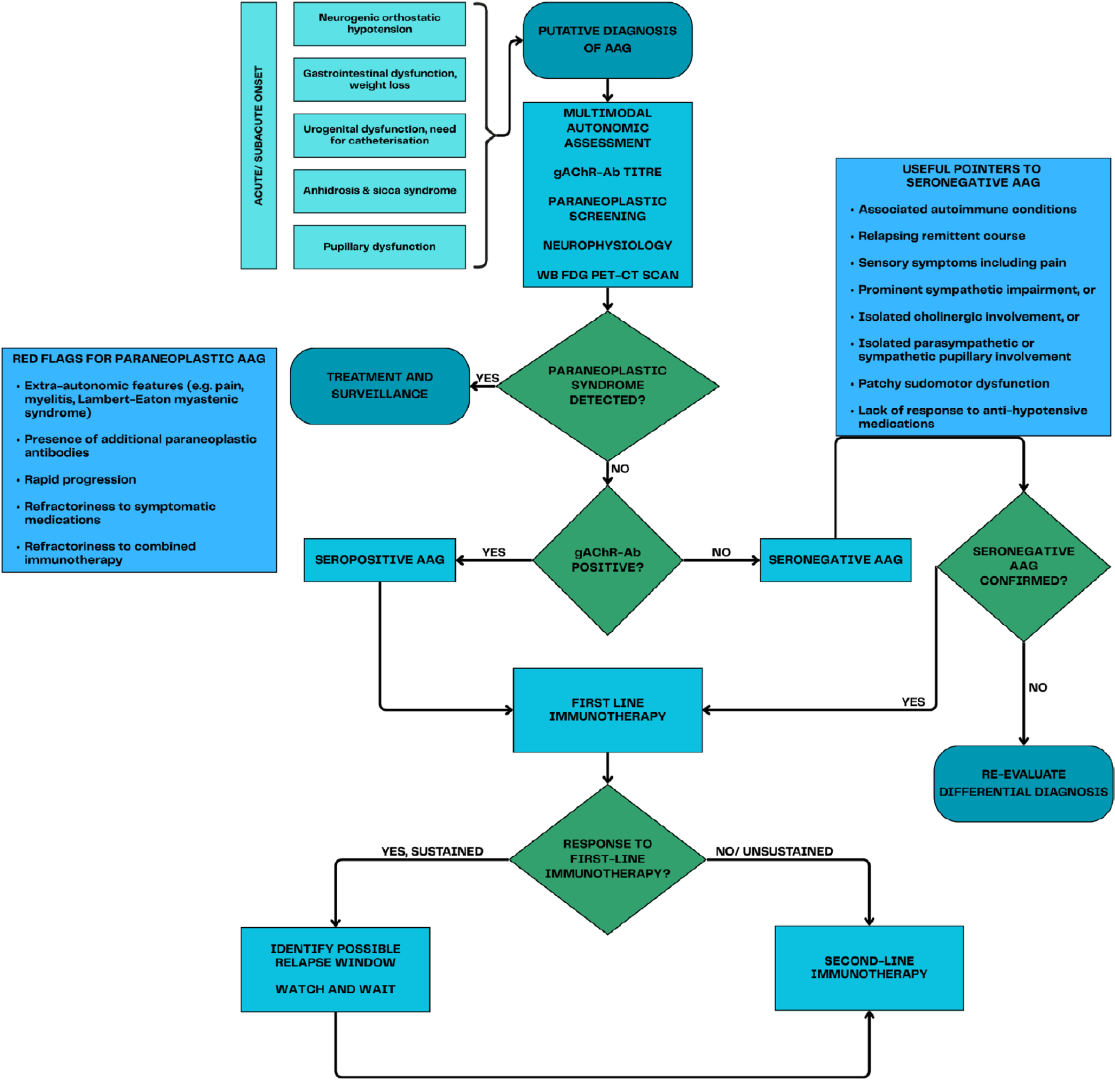
AAG pathway.

Our workup and therapy pathway is described in Figure 1. Subjects presenting with an acute, subacute, or chronic onset of multi‐domain autonomic symptoms and evidence of autonomic failure are considered for a diagnosis of putative AAG, as previously defined [25]. They undergo a baseline multimodal autonomic assessment, which includes cardiovascular autonomic function testing, 24 h ambulatory blood pressure monitoring, quantification of plasma catecholamine levels, gAChR‐Ab levels, pupillometry, Schirmer's test, unstimulated saliva production test, uroflowmetry, post‐void bladder ultrasound, and COMPASS‐31 questionnaires. Laboratory blood tests for causes of acquired neuropathy, paraneoplastic antibodies, neurophysiology, a whole‐body FDG PET/CT scan, or a combination of chest X‐ray/CT scan, abdominopelvic ultrasound/CT scan, and testicular ultrasound in males are also obtained.

Once a diagnosis of seropositive AAG is confirmed and other causes of autonomic failure are excluded, patients are started on a first line treatment with either PLEX 3 cycles over 5 days every 4–6 weeks, or IVIG 2 g/Kg 3 cycles over 4–5 days every 4 weeks. The multimodal autonomic assessment is repeated 2 weeks post‐PLEX or post‐IVIG.

We defined response to treatment as a combination of (1) symptomatic improvement as suggested by clinical history, use of symptomatic medications (mostly vasopressors and laxatives), and/or COMPASS‐31 scores; associated with (2) objective evidence of improvement in autonomic function measurements such as ΔSBP on AST and/or HUT, OIR, respiratory sinus arrhythmia (RSA) Med, VM profile, PVR volume, use of catheter, pupillary measures, and the gAChR‐Ab titre.

If there is a documented response to PLEX or IVIG, a watch‐and‐wait approach is used to determine the need for ongoing therapy and frequency of treatment required, as previously described for chronic inflammatory peripheral neuropathies [32]. In case of a relapse, patients repeat the multimodal autonomic biomarker assessment and receive one further cycle of IVIG, which is subsequently given at regular intervals determined by the time to relapse. Once a timeframe of response to IVIG is established, patients are started on a second‐line, longer‐term immunosuppression, usually beginning with prednisolone (1 mg/kg, maximum of 60 mg/day) for 6 weeks, then tapered down by 10 mg/month and imbricated with a steroid‐sparing agent, such as mycophenolate mofetil or azathioprine.

Patients are monitored clinically every 3 months and with repeat multimodal autonomic assessments every 6–12 months. If refractory to treatment, or if there are other red flags for a paraneoplastic disease (such as dual antibodies), they undergo long‐term surveillance with a whole‐body FDG PET/CT scan every 2 years (Figure 1).

### 2.6 Statistical Analysis

GraphPad Prism version 9 was used for statistical analysis. Data were tested for normality by the Shapiro–Wilk test and are provided as median (interquartile range [IQR]) as nonparametric. Baseline and follow‐up parameters after immunotherapy are provided as median change (95% CI) and were compared with Mann–Whitney U tests. Spearman's rank correlations were used to assess correlations between autonomic function measures and COMPASS‐31 scores as appropriate. Two‐sided *p* < 0.05 was considered significant.

## 3 Results

### 3.1 Patients

Between 2005 and August 2025, 22 patients (8 women, 36%) received a diagnosis of AAG, of which 18/22 (82%) had a positive gAChR‐Ab titre. Longitudinal, repeated quantitative multimodal outcome measures of autonomic function, gAChR‐Ab titres, COMPASS‐31 scores, and medication use pre‐ and post‐treatment were available for 16 patients (6 women, 37%) with seropositive AAG. Only these 16 patients were included in this work. Outcome measures were collected at baseline (usually pre‐PLEX), post‐PLEX, pre‐ and post‐IVIG, and pre‐ and post‐oral immunotherapy (either prednisolone, steroid‐sparing agents, or a combination of both).

Median age at disease onset was 50 (IQR 40–58) years. The median delay between disease onset and first autonomic assessment was 2 (IQR 1–4) years. Median disease duration was 8 (IQR 6–11) years from symptoms' onset to last available contact. Five patients (31%) died during follow‐up; 4 of these had an oncological diagnosis, and the cause of death for 1 patient remained unknown.

5/16 (31%) patients had other autoimmune conditions, including hypothyroidism (*n* = 4), inflammatory bowel disease (*n* = 2), psoriasis (*n* = 1), idiopathic urticaria (*n* = 1), Addison's disease (*n* = 1), pernicious anaemia (*n* = 1), and alopecia totalis (*n* = 1). 5/16 (31%) patients had an oncological diagnosis, including large intestine adenocarcinoma (*n* = 1), non‐small cell lung cancer (*n* = 1), chronic lymphocytic leukaemia (*n* = 1), and ovarian teratoma (*n* = 1). Details are available in (Table 1).

**TABLE 1:** Demographics, clinical features, and overall overview of disease‐modifying treatment.

Parameter	Seropositive AAG patients (16)		
Sex (F)	6 (37%)		
Age at onset (years)	50 (40–58)		
Onset to AFT delay (years)	2 (1–4)		
Disease duration (as of Aug 24)	8 (6–11)		
Deaths	4 (25%)		
Seropositive	16 (100%)		
Autoimmune history (pts)	6 (37%) Hypothyroidism (4) Crohn's disease/ulcerative colitis (2) Psoriasis (1) Addison's disease (1) Alopecia totalis (1) Idiopathic urticaria (1) Coeliac disease (1) Pernicious anaemia (1)		
Oncological history	5 (31%) Ovarian teratoma (1) Large intestine adenocarcinoma (1) Non‐small cell lung carcinoma (1) Lung cancer (1) Chronic lymphocytic leukaemia (1)		
	No. cycles	Duration (weeks)	AE
PLEX (16)	3 (3–5)	32 (24–73)	5 3 DVT 1 PE 1 TLOC
IVIg (10)	3 (3–8)	39 (10–134)	0
Prednisolone (11)	—	64 (26–90)	2 1 lower limb oedema, weight gain, oral thrush, low mood 1 low mood, vivid dreams, hair loss, anxiety
	Type of medication	Duration (weeks)	AE
Immunotherapy (11)	7 MMF 1 AZA 1 MMF + MTX 1 MMF + RTX 1 CLB, RTX, ara‐C, CP	202 (78–241)	1 anaemia

*Note:* Data are presented as median (IQR) or as total no. (%) of patients.

Abbreviations: AE, adverse event; AFT, autonomic function testing; ara‐C, cytarabine; AZA, azathioprine; CLB, chlorambucil; CP, cyclophosphamide; DVT, deep venous thrombosis; IVIg, intravenous immunoglobulins; MMF, mycophenolate; MTX, methotrexate; PE, pulmonary embolism; PLEX, plasma exchange; RTX, rituximab; TLOC, transient loss of consciousness.

### 3.2 Baseline Cardiovascular Autonomic Function Tests

At baseline autonomic function testing, all patients had evidence of widespread cardiovascular autonomic failure, as described in Table 2.

### 3.3 Disease‐Modifying Treatment

**FIGURE 2:**
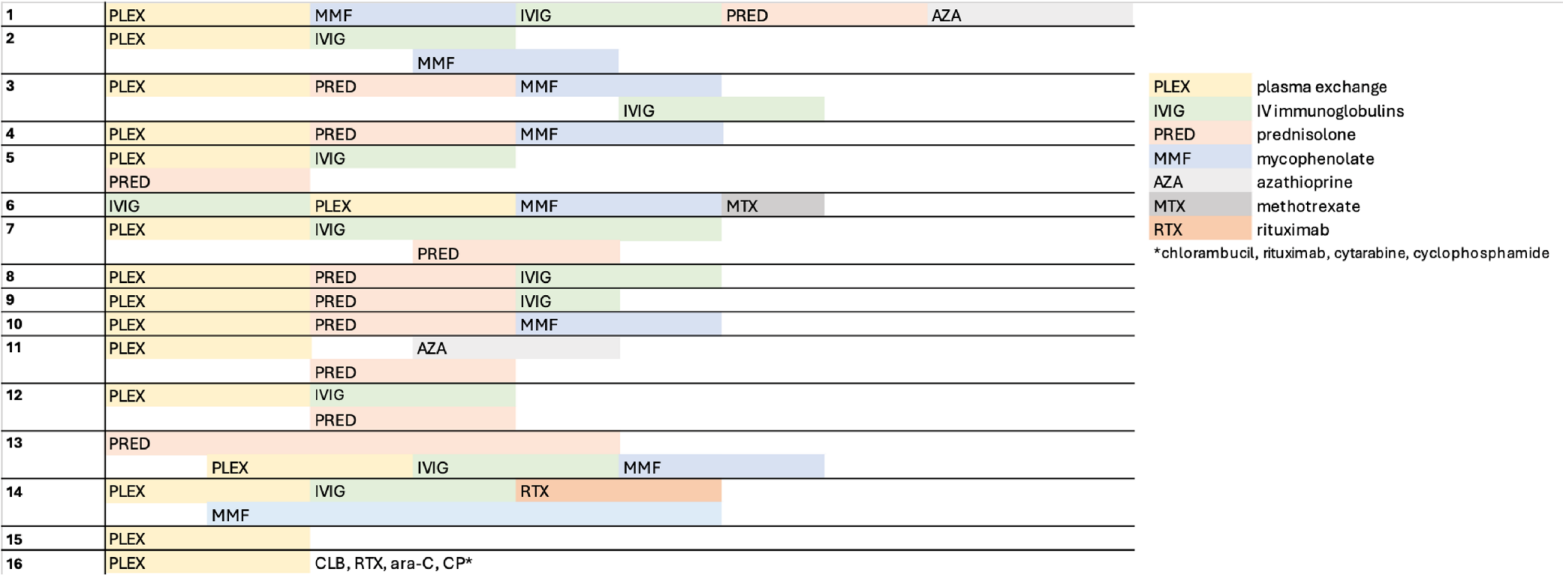
Treatment timeline.

16/16 patients (100%) received at least one combination of first line disease‐modifying treatment with PLEX and/or IVIG and second line therapy with steroids and steroid‐sparing agents (Figure 2). Median delay between disease onset and initiation of immune treatment was 3 (IQR 2–4) years. Median treatment duration was 6 (4–8) years. As of August 2025, all alive patients were still receiving maintenance immunotherapy.

16/16 (100%) patients received 3 (3–5) PLEX cycles over 32 (IQR 24–73) weeks. There were 5 adverse events leading to its discontinuation (*n* = 3 deep vein thrombosis, *n* = 1 saddle pulmonary embolism, *n* = 1 transient loss of consciousness). 10/16 (62%) patients received 2 (IQR 1–4) IVIG cycles, over 39 (IQR 10–134) weeks. 11/16 (69%) patients received high‐dose prednisolone for 64 (IQR 26–90) weeks. This was discontinued prematurely in 2 patients because of side effects (*n* = 1 lower limb oedema, weight gain, oral thrush, low mood; *n* = 1 low mood, vivid dreams, hair loss, anxiety). 11/16 (69%) patients proceeded to long‐term steroid‐sparing agents (*n* = 7 mycophenolate, *n* = 1 azathioprine, *n* = 1 mycophenolate and methotrexate, *n* = 1 mycophenolate and rituximab, *n* = 1 chlorambucil, rituximab, cytarabine, cyclophosphamide) for 202 (78–241) weeks. 1 patient developed anaemia while taking mycophenolate, which was discontinued (Table 2).

**TABLE 2:** Comparison of assessments before and after whole treatment.

Parameter (*n*)	Pre‐treatment	Post‐treatment	Change^a^	*p*
gAChR Ab (pmol/L)	724 (454–886)	203 (132–266)	−350 (−652–−241)	0.005*
Cardiovascular autonomic biomarkers (16)				
Mean supine SBP (mmHg)	139 (114–151)	139 (123–149)	11 (−27–41)	0.32
Mean supine DBP (mmHg)	83 (70–91)	76 (71–90)	1 (−18–12)	0.73
Mean supine HR (bpm)	68 (64–75)	69 (61–71)	−0,5 (−8–4)	0.87
Lowest SBP on HUT (mmHg)	61 (55–83)	83 (75–112)	22 (4–50)	0.001**
Lowest DBP on HUT (mmHg)	42 (39–48)	54 (45–69)	7 (−3–37)	0.003**
Last HR on HUT (bpm)	70 (66–76)	71 (67–79)	1 (−1–7)	0.28
ΔSBP (mmHg)	70 (83–48)	41 (64–36)	22 (−13–57)	0.05*
ΔDBP (mmHg)	41 (49–27)	21 (45–12)	10 (−3–32)	0.12
ΔHR (bpm)	0 (0–1)	1,5 (0,7–5)	1 (−1 −5)	0.02*
Time on tilt (min)	3 (1–9)	10 (10–10)	6 (0–9)	0.001**
OIR	20 (44–7)	4 (9–4)	16 (0–40)	0.003**
E/I ratio	2 (0–4)	4 (2–5)	0,5 (−2−5)	0.40
VM ratio	1,04 (1,00–1,06)	1,09 (1,00–1,18)	0,01 (−0,06−0,24)	0.14
Pupillometry (8)				
Pupillary light response, %				
R	13 (8–19)	19 (8–32)	34 (−20–343)^b^	0.05*
L	14 (5–23)	24 (13–37)	49 (−15–572)^b^	0.05*
Uroflowmetry (7)				
PVR volume, ml	222 (16–392)	90 (27–300)	−87 (−302–127)	0.40
Void time, s	70 (45–86)	50 (28–116)	−1 (−43−45)	0.95
COMPASS‐31 (16)				
Total score	54 (41–66)	46 (36–56)	−13 (−17−5)	0.09
Orthostatic	30 (20–32)	24 (18–28)	−8 (−10–−2)	0.08
Vasomotor	0 (0–0)	0 (0–2)	0 (0–2)	0.19
Secretomotor	7 (4–9)	9 (6–10)	0 (−6–7)	0.71
Gastrointestinal	11 (9–12)	12 (4–13)	−4 (−7–3)	0.84
Bladder	4 (2–7)	4 (2–5)	−1 (−3–0)	0.63
Pupil	2 (2–3)	2 (0–3)	−1 (−1–0)	0.32

*Note:* Data are presented as median (IQR).

Abbreviations: DBP, diastolic blood pressure; E/I, exhalation‐inhalation ratio; gAChR‐Ab, ganglionic acetylcholine receptor antibody; HR, heart rate; HUT, head‐up tilt; OIR, orthostatic intolerance ratio; PVR, post‐void residual; SBP, systolic blood pressure; VM, Valsalva manoeuvre.

^a^ As mean or median change (95% CI) according to difference distribution.

^b^ As % change (95% CI), */** statistically significant.

#### 3.3.1 Overall Treatment Response to Combined Long‐Term Disease‐Modifying Treatment

After combined immunotherapy (mean delay to post‐treatment testing 40 [13–185] weeks), there were significant and sustained improvements in measures of adrenergic function and orthostatic tolerance (pre‐ vs. post‐treatment ΔSBP change 22 [−13–57] mmHg, *p* = 0.05, time tolerated on tilt change 6 [0–9] min, *p* = 0.001, OIR change 16 [0–40], *p* = 0.003). NOH recovered in four patients. Pupillary reaction to light improved (R 34 (−20–343) %, L 49 (−15–572) %, *p* = 0.05). There were significant reductions in the gAChR‐Ab titre (−350 (−652 to −241) pmol/L, *p* = 0.005). While statistically not significant, likely due to small sample size, there was a clinical improvement in measures of urinary flow (PVR volume, −87 [−302 to −127] mL). COMPASS‐31 total and subdomain scores showed a consistent trend toward improvement post‐treatment (Table 2).

#### 3.3.2 Treatment Response to First‐Line Treatment With PLEX and IVIG

Repeated measures of cardiovascular autonomic function were available for all patients receiving PLEX and IVIG. Patients were reassessed after a median of 17 (12–22) days following PLEX. There were clinical improvements in measures of adrenergic function such as ΔSBP, time tolerated on tilt, and the OIR. NOH resolved in 1 patient. There was a clinically relevant reduction in the gAChR‐Ab titres (Table S1).

After a median of 22 (18–24) days post‐IVIG, there were significant improvements in the time tolerated on tilt (5 [0–10] min, *p* = 0.006) and the OIR (9 [−2–103], *p* = 0.02). NOH resolved in 2 patients. There was a reduction in the gAChR‐Ab titres (Table S1).

Overall, these changes were transient, with most patients needing repeated cycles of PLEX and/or IVIG (median 3 [3–5] and 3 [3–8] cycles respectively). All patients required second‐line immunotherapy with steroids or steroid‐sparing agents.

### 3.4 Treatment Response and Residual Burden

**FIGURE 3:**
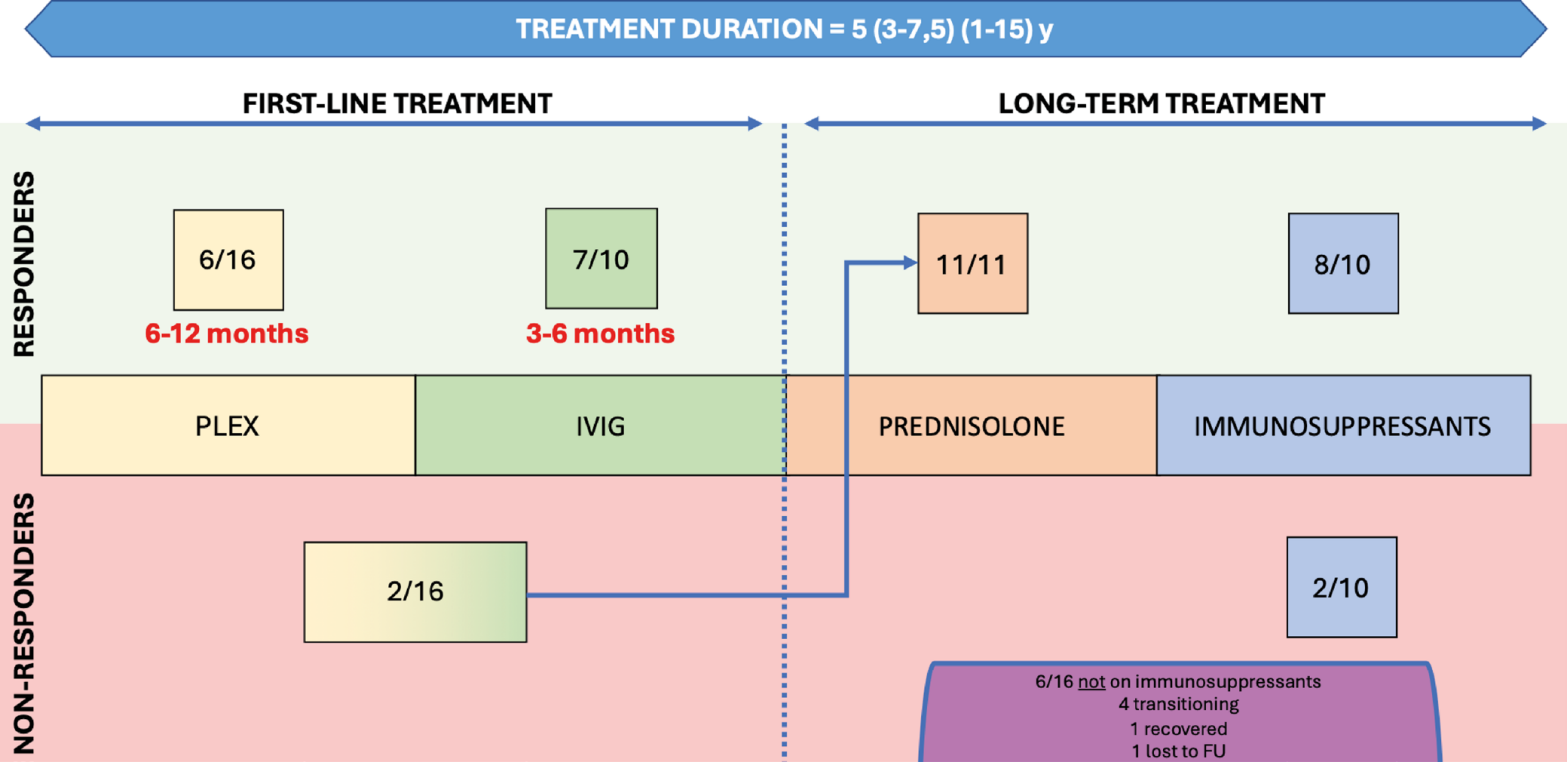
Responders vs. non‐responders.

According to our definition of treatment response, we identified 2 patients who did not respond to first‐line treatment with either PLEX or IVIG but were successfully treated with prednisolone. Another 2 patients who showed a positive temporary response to PLEX and/or IVIG were further stabilised with prednisolone but relapsed under steroid‐sparing immunotherapy (Figure 3).

Despite functional improvement on autonomic outcome measures and partial subjective improvement, most patients remained on a combination of vasopressors due to the residual severity of nOH. Laxative use remained unchanged. 4/8 (50%) patients were able to discontinue catheterisation after treatment (Table S3).

### 3.5 Correlations

Correlations between measures of autonomic function, gAChR‐Ab titres and COMPASS‐31 scores were drawn. High gAChR‐Ab titres correlated with the severity of cardiovascular autonomic failure at disease onset, as measured by the minimum SBP and OIR on HUT pre‐treatment (Spearman r = −0,5 [−0,8–0,03], *p* = 0.05 and Spearman *r* = 0,7 [0,3–0,9], *p* = 0.003 respectively), as well as with uroflowmetry measures (difference in time to maximum flow and in void time, Spearman *r* = 0,8, *p* = 0.04 and Spearman *r* = 0,9, *p* = 0.006 respectively). There were no correlations between antibody titres and autonomic function measures post‐treatment.

The COMPASS‐31 orthostatic subdomain scores correlated with adrenergic function measures both at baseline and after treatment (ΔSBP on HUT pre‐treatment, Spearman r = −0,8, *p* = 0.01; OIR post‐treatment, Spearman r = −0,7, *p* = 0.03; minimum SBP on HUT post‐treatment, Spearman *r* = 0, 7 *p* = 0.02). No correlations with measures of urinary or pupillomotor function were found.

## 4 Discussion

We presented the long‐term treatment outcomes of our cohort of seropositive AAG patients, followed up for up to 10 years with repeated assessments of autonomic function at specific timepoints along their treatment pathway. Our data answers several questions and provides useful lessons for future clinical implementations.

All patients had objective evidence of severe and widespread autonomic failure in multiple domains at disease onset. Only one patient demonstrated a monophasic illness. Most patients had relapsing, remitting, or chronic disease.

30% of our patients were considered to have a paraneoplastic form of AAG, which is a ratio in line with previous literature. The median time between the onset of autonomic failure and the oncological diagnosis was 3 years, with a range of up to 9 years, which is significantly longer than previously reported for most neurological paraneoplastic syndromes [33, 34]. It may be argued whether some of the oncological diagnoses were incidental. In our clinical experience, a deterioration after previously well‐controlled disease should prompt repeat screening for possible malignancy. Although the European Federation of Neurological Societies Task Force states that if primary screening is negative, screening should be repeated after 3–6 months and then every 6 months up until 4 years [35], we suggest prolonging the screening window in these patients.

In terms of initial diagnostic workup and monitoring, we strongly advocate for the use of a quantitative multimodal autonomic assessment in AAG, as it provides a comprehensive evaluation of autonomic function across multiple domains. This approach has proven valuable in capturing baseline impairments, guiding immunotherapy, and monitoring treatment response. Given that AAG is a patchy disorder affecting several autonomic domains with varying severity, it is imperative to employ a broad range of tools capable of detecting these diverse abnormalities. While there continues to be no international guideline on the diagnosis and treatment of AAG, the pathway presented in this work represents our current clinical practice recommendation after decades of managing patients with this rare disease as a national referral centre.

Response to treatment is challenging to define in a multisystemic and fluctuating disease such as AAG, and there is no consensus available in the previous literature [20, 36]. We suggest that a disease‐specific score including subjective and quantitative functional measures should be developed as suitable outcome measures to assess response. We confirmed once again that gAChR‐Ab levels correlated with the severity of autonomic failure at disease onset, as in previous reports [9, 37], but did not capture disease progression or response to treatment [4].

Two patients did not respond to first‐line treatment with either PLEX or IVIG, but were successfully treated with prednisolone, according to our definition. Another 2 patients showed a positive temporary response to PLEX and/or IVIG, were further stabilised with prednisolone, but relapsed under steroid‐sparing immunotherapy. In our experience, PLEX and IVIG only provided a temporary benefit, and the latter were generally better tolerated than PLEX, which was associated with serious adverse events. In newly diagnosed patients, we currently use IVIG as the first‐line treatment following the update in NHS England commissioning guidance. We have now largely discontinued the use of plasma exchange (PLEX), preferring IVIG both for initial therapy and, when necessary, as a rescue treatment during relapses.

Steroids and steroid‐sparing agents provided a more solid and long‐lasting therapeutic effect in most of our patients. These findings are in line with previous reports and highlight that a combined, step‐wise multi‐agent immunomodulatory approach may be necessary to satisfactorily treat AAG [20, 22, 38]. Nonetheless, residual autonomic impairment remained in all but 2 patients who had a monophasic disease course, as mirrored by the continued use of vasopressor medications and laxatives. Future therapeutic interventions include the use of rituximab on a larger scale.

This study has some limitations. Assessment of gastrointestinal and sudomotor function was missing, with the intention to be added in future works. Our screening for paraneoplastic syndromes was originally limited to a whole‐body PET‐CT scan every 2 years. This has now been changed to mirror the recommendations made by the European Federation of Neurological Societies Task Force. As the largest autonomic unit in the United Kingdom, our centre was well placed to investigate this rare disorder. Nevertheless, patients with milder manifestations may have been managed exclusively by local services and therefore not referred to our institution. Consequently, a referral bias may have resulted in our cohort being skewed toward individuals with more severe disease.

## 5 Conclusions

This study presents the long‐term outcomes of a cohort of seropositive AAG patients followed up to 10 years, demonstrating that while most patients experience a relapsing–remitting or progressive course, sustained improvement is achievable with a stepwise, multi‐agent immunotherapy approach. Quantitative multimodal autonomic assessment proves valuable in defining baseline impairment, guiding treatment, and monitoring response to long term immunotherapy. Our findings support the preferential use of IVIG as both first line and rescue therapy, given its more favourable tolerability profile compared with PLEX at the time this study was conducted. In centres where IVIG availability is limited, PLEX remains a reliable option for inducing initial disease remission. We also strongly advocate for prolonged malignancy surveillance and the development of disease‐specific outcome measures to capture treatment response more accurately. In the absence of controlled trial data, management of autoimmune autonomic ganglionopathy must be tailored to each patient's clinical profile and longitudinal response to immunotherapy.

## Author Contributions

G.C. and V.I. contributed to the conception and design. G.C., S.K., F.B., L.W., G.T.I., and P.M. contributed to acquisition and analysis of data. G.C. and V.I. contributed to drafting the text and preparing the figures.

## Funding

Dr. V Iodice is supported by National Institute for Health Research University College London Hospitals Biomedical Research Centre. Dr. V Iodice has received honoraria from Theravance Biopharma not related to this work. Dr. S Koay was supported by the Guarantors of Brain Entry Fellowship.

## Conflicts of Interest

The authors declare no conflicts of interest.

## Supporting information


**Table S1:** Comparison of assessments before and after PLEX‐AFT delay = 17 [12–22] days. PLEX = plasma exchange; gAChR Ab = ganglionic acetylcholine receptor antibody; SBP = systolic blood pressure; DBP = diastolic blood pressure; HR = heart rate; HUT = head‐up tilt; OIR = orthostatic intolerance ratio; E/I exhalation‐inhalation ratio; VM = Valsalva manoeuvre. Data are presented as median (IQR), ^†^as mean or median change (95% CI) according to difference distribution.
**Table S2:** Comparison of assessments before and after IVIG (IVIG‐AFT delay = 22 [18–24] days). IVIg = intravenous immunoglobulins; gAChR Ab = ganglionic acetylcholine receptor antibody; SBP = systolic blood pressure; DBP = diastolic blood pressure; HR = heart rate; HUT = head‐up tilt; OIR = orthostatic intolerance ratio; E/I exhalation‐inhalation ratio; VM = Valsalva manoeuvre; *R* = right; L = left. Data are presented as median (IQR), ^†^as mean or median change (95% CI) according to difference distribution, ^‡^as % change (95% CI).
**Table S3:** Number of patients on symptomatic medications and catheterization. PLEX = plasma exchange; PRED = prednisolone; ImmTx = steroid‐sparing immunotherapy; ISC = intermittent self‐catheterisation; SPC = suprapubic catheterisation. Data are presented as total number of patients.

## Data Availability

The data that support the findings of this study are available from the corresponding author, upon reasonable request.
