# Supplementary material for: Long‐Term Efficacy of Immunotherapy in Autoimmune Autonomic Ganglionopathy—A 10‐Year Follow Up Study
Source: Ann Clin Transl Neurol. 2026 May 15:10.1002/acn3.70421. Online ahead of print. doi: 10.1002/acn3.70421 (PMC13394140; doi:10.1002/acn3.70421)
Supplement: Supplementary file 1 — Table S1: Comparison of assessments before and after PLEX‐AFT delay = 17 [12–22] days. PLEX = plasma exchange; gAChR Ab = ganglionic acetylcholine receptor antibody; SBP = systolic blood pressure; DBP = diastolic blood pressure; HR = heart rate; HUT = head‐up tilt; OIR = orthostatic intolerance ratio; E/I exhalation‐inhalation ratio; VM = Valsalva manoeuvre. Data are presented as median (IQR), †as mean or median change (95% CI) according to difference distribution. Table S2: Comparison of assessments before and after IVIG (IVIG‐AFT delay = 22 [18–24] days). IVIg = intravenous immunoglobulins; gAChR Ab = ganglionic acetylcholine receptor antibody; SBP = systolic blood pressure; DBP = diastolic blood pressure; HR = heart rate; HUT = head‐up tilt; OIR = orthostatic intolerance ratio; E/I exhalation‐inhalation ratio; VM = Valsalva manoeuvre; R = right; L = left. Data are presented as median (IQR), †as mean or median change (95% CI) according to difference distribution, ‡as % change (95% CI). Table S3: Number of patients on symptomatic medications and catheterization. PLEX = plasma exchange; PRED = prednisolone; ImmTx = steroid‐sparing immunotherapy; ISC = intermittent self‐catheterisation; SPC = suprapubic catheterisation. Data are presented as total number of patients. [file ACN3-9999-0-s001.docx]

| *Parameter (n)* | Pre-PLEX | Post-PLEX | Change^†^ | *p* value |
| --- | --- | --- | --- | --- |
| gAChR Ab (pmol/L) | 588 (449 – 963) | 393 (154 – 871) | -255 (-702 – 422) | 0.23 |
| *Quantitative autonomic biomarkers (16)* | | | | |
| Mean supine SBP (mmHg) | 141 (120 – 177) | 124 (112 – 177) | -1 (-42 – 37) | 0.73 |
| Mean supine DBP (mmHg) | 87 (76 – 94) | 79 (67 – 99) | -7 (-27 – 7) | 0.18 |
| Mean supine HR (bpm) | 70 (64 – 79) | 72 (62 – 78) | 1 (-12 – 12) | 0.93 |
| Lowest SBP on HUT (mmHg) | 84 (54 – 106) | 71 (53 – 107) | 1 (-20 – 15) | 0.70 |
| Lowest DBP on HUT (mmHg) | 46 (39 – 76) | 57 (40 – 81) | 8 (1 – 14) | 0.01* |
| Last HR on HUT (bpm) | 72 (64 – 79) | 71 (43 – 84) | 4 (-23 -15) | 0.76 |
| ΔSBP (mmHg) | 66 (90 – 36) | 49 (117 – 17) | 7 (-45 – 50) | 0.90 |
| ΔDBP (mmHg) | 41 (53 – 18) | 21 (59 – 5) | 12 (-6 – 41) | 0.10 |
| ΔHR (bpm) | 0 (-1 – 6) | 1 (-25 – 16) | -1 (-25 – 10) | 0.73 |
| Time on tilt (min) | 4 (1 – 10) | 10 (3 – 10) | 3 (-3 – 8) | 0.11 |
| OIR | 20 (4 – 66) | 7 (2 – 37) | 13 (-22 – 57) | 0.20 |
| E/I ratio | 3 (0 – 30) | 4 (0 – 7) | 0 (-26 – 4) | 0.99 |
| VM ratio | 1,05 (0,8 – 1,3) | 1,11 (0,9 – 1,3) | 0 (0 – 0) | 0.68 |

Supplementary Table 1. Comparison of assessments before and after PLEX (PLEX-AFT delay = 17 [12 – 22] days]. PLEX = plasma exchange; gAChR Ab = ganglionic acetylcholine receptor antibody; SBP = systolic blood pressure; DBP = diastolic blood pressure; HR = heart rate; HUT = head-up tilt; OIR = orthostatic intolerance ratio; E/I exhalation-inhalation ratio; VM = Valsalva manoeuvre. Data are presented as median (IQR), ^†^as mean or median change (95% CI) according to difference distribution.

| *Parameter (n)* | Pre-IVIG | Post-IVIG | Change^†^ | *p* value | |
| --- | --- | --- | --- | --- | --- |
| gAChR Ab (pmol/L) | 342 (104 – 597) | 141 (110 – 231) | -73 (-732 – 53) | 0.33 | |
| *Cardiovascular autonomic biomarkers (10)* | | | | | |
| Mean supine SBP (mmHg) | 143 (122 – 171) | 138 (124 – 187) | 7 (-31 – 37) | 0.66 | |
| Mean supine DBP (mmHg) | 82 (76 – 88) | 83 (76 – 93) | 2 (-9 – 32) | 0.43 | |
| Mean supine HR (bpm) | 68 (66 – 70) | 66 (61 – 75) | -1 (-7 – 10) | 0.66 | |
| Lowest SBP on HUT (mmHg) | 76 (47 – 83) | 91 (77 – 113) | 13 (-4 – 83) | 0.07 | |
| Lowest DBP on HUT (mmHg) | 47 (19 – 54) | 57 (46 – 78) | 8 (-46 – 64) | 0.11 | |
| Last HR on HUT (bpm) | 69 (62 – 77) | 69 (62- 75) | -0,5 (-6 – 14) | 0.59 | |
| ΔSBP (mmHg) | 75 (108 – 44) | 40 (115 – 13) | 14 (-36 – 66) | 0.14 | |
| ΔDBP (mmHg) | 32 (64 – 23) | 33 (62 – 0,5) | 12 (-52 – 56) | 0.29 | |
| ΔHR (bpm) | 0,6 (-3 – 6) | 4 (1 – 5) | 0,6 (-4 – 8) | 0.41 | |
| Time on tilt (min) | 4,5 (1 – 9) | 10 (10 – 10) | 5 (0 – 10) | 0.006** | |
| OIR | 15 (3 – 26) | 4 (1 – 11) | 9 (-2 – 103) | 0.02* | |
| E/I ratio | 2 (0 – 5) | 3 (0,5 – 5) | -0,5 (-3 – 11) | 0.85 | |
| VM ratio | 1,04 (0,97 – 1,10) | 1,12 (1,00 – 1,27) | 0,07 (-0,03 – 0,42) | 0.08 | |
| *Pupillometry (6)* | | | | | |
| Pupillary light response, %  R  L | 17 (13 – 20)  16 (10 – 28) | 16 (12 – 30)  20 (13 – 27) | 9 (-44 – 65)^‡^  19 (-31 – 40)^‡^ | | 0.40  0.29 |

Supplementary Table 2. Comparison of assessments before and after IVIG (IVIG-AFT delay = 22 [18 – 24] days). IVIg = intravenous immunoglobulins; gAChR Ab = ganglionic acetylcholine receptor antibody; SBP = systolic blood pressure; DBP = diastolic blood pressure; HR = heart rate; HUT = head-up tilt; OIR = orthostatic intolerance ratio; E/I exhalation-inhalation ratio; VM = Valsalva manoeuvre; R = right; L = left. Data are presented as median (IQR), ^†^as mean or median change (95% CI) according to difference distribution, ^‡^as % change (95% CI).

|  | *Pre-PLEX*  *(14 pts)* | *Post-PLEX*  *(14 pts)* | *Pre-IVIg*  *(8 pts)* | *Post-IVIg*  *(8 pts)* | *Pre-PRED/ImmTx*  *(8 pts)* | *Post-PRED/ImmTx*  *(8 pts)* | *Pre-treatment*  *(16 pts)* | *Post-treatment*  *(16 pts)* |
| --- | --- | --- | --- | --- | --- | --- | --- | --- |
| *Anti-Hypotensives* | 13 | 13 | 9 | 9 | 8 | 8 | 15 | 13 |
| *Fludrocortisone* | 11 | 11 | 8 | 8 | 6 | 7 | 13 | 11 |
| *Midodrine* | 10 | 10 | 8 | 8 | 5 | 6 | 10 | 10 |
| *Octreotide* | 1 | 1 | 1 | 2 | 1 | 2 | 1 | 3 |
| *Ephedrine* | 2 | 2 | 1 | 1 | 0 | 1 | 2 | 2 |
| *Desmopressin* | 2 | 0 | 0 | 0 | 0 | 0 | 2 | 0 |
| *Pyridostigmine* | 1 | 1 | 1 | 2 | 1 | 1 | 1 | 2 |
| *Laxatives* | 6 | 6 | 4 | 6 | 4 | 5 | 7 | 8 |
| *Antimuscarinics/ beta3 agonists* | 0 | 1 | 0 | 0 | 1 | 0 | 0 | 0 |
| *PDE5 inhibitors* | 0 | 0 | 0 | 0 | 0 | 1 | 0 | 1 |
| *ISC* | 7 | 6 | 3 | 3 | 5 | 3 | 7 | 4 |
| *SPC* | 0 | 0 | 1 | 0 | 0 | 0 | 1 | 0 |

Supplementary Table 3. Number of patients on symptomatic medications and catheterization. PLEX = plasma exchange; PRED = prednisolone; ImmTx = steroid-sparing immunotherapy; ISC = intermittent self-catheterisation; SPC = suprapubic catheterisation. Data are presented as total no. of patients.
